# Exploratory dose modeling of hemoadsorption in pediatric septic shock

**DOI:** 10.1186/s40635-026-00933-1

**Published:** 2026-06-22

**Authors:** Gabriella Bottari

**Affiliations:** Pediatric Intensive Care Unit, Children Hospital Bambino Gesù, IRCCS, Largo Sant’Onofrio 4, Rome, 00162 Italy

**Keywords:** Sepsis, septic shock, hemoadsorption, Pediatric septic shock, Blood purification, Cytokines

## Abstract

**Background:**

Clinical outcomes of hemoadsorption (HA) in sepsis remain uncertain despite demonstrated biological efficacy in in vivo models. Persistent knowledge gaps concern both patient selection—now increasingly interpreted through biological phenotypes—and optimal device application. In recent years, this latter aspect has been conceptualized as “dose,” yet HA dosing remains poorly defined and inconsistently applied across age groups. Interestingly, pediatric studies have reported more favorable outcomes than adult cohorts. We therefore investigated the concept of dose in pediatric septic shock to elucidate mechanistic determinants of treatment efficacy.

**Methods:**

We retrospectively analyzed 25 children with septic shock treated with CytoSorb^®^ combined with continuous renal replacement therapy. HA dose was expressed as Amount of Blood Purified (ABP, L/kg). Kinetic simulations incorporating staged extraction fractions were performed to model progressive cartridge saturation. Cytokine removal was modeled using log-transformed IL-6. Multivariable linear regression models assessed the relationship between ABP, baseline cytokine concentration, and cytokine reduction.

**Results:**

Median ABP was 14.4 (9.7–21.6) L/kg, substantially exceeding previously reported adult thresholds. No linear association was observed between ABP and vasopressor reduction. IL-6 reduction was strongly associated with baseline cytokine concentration (R² = 0.79, 95%CI= [-1.08; -0.62], *p* < 0.001), while ABP was not independently predictive.

**Conclusions:**

In this exploratory pediatric cohort, hemoadsorption during CRRT was associated with high blood-purified-per-kilogram exposure and marked cytokine reduction. These findings suggest that, beyond a certain exposure level, mediator removal may become predominantly concentration-driven rather than dose-limited, supporting further investigation of dosing strategies based on inflammatory burden.

**Supplementary Information:**

The online version contains supplementary material available at 10.1186/s40635-026-00933-1.

## Background

Sepsis is characterized by a dysregulated host response to infection, in which excessive and imbalanced cytokine release contributes to organ dysfunction and mortality. Extracorporeal hemoadsorption (HA) using polymer-based cartridges (e.g., divinylbenzene resins) has been proposed as a strategy to modulate the “cytokine storm” through non-selective removal of circulating inflammatory mediators. Despite a strong pathophysiological rationale, clinical results remain inconsistent. Recent systematic reviews and meta-analyses have failed to demonstrate a consistent mortality benefit [1]. Sepsis, however, is a biologically heterogeneous syndrome with dynamic and patient-specific inflammatory trajectories. Applying HA in an unselected, uniform manner may therefore obscure potential benefits in specific subgroups [2].

In an in vivo human endotoxemia model, Jansen et al. demonstrated that a divinylbenzene-based cartridge effectively removes circulating cytokines in a concentration-dependent fashion following lipopolysaccharide stimulation [3]. These data confirm that cytokine adsorption follows predictable kinetic principles, with clearance proportional to circulating levels.

Sorbent-based adsorption devices follow non-linear, saturation-dependent kinetics, characterized by high initial clearance followed by progressive decline as binding sites become occupied. Therefore, delivered dose cannot be interpreted solely as cumulative blood exposure, but must be contextualized within the dynamic interplay between extraction efficiency and mediator concentration [4].

The key knowledge gap at the bedside may therefore concern not only whether to use hemoadsorption, but how to dose and manage it. The first clinical application of HA in 2019—in a case of cytokine release syndrome (CRS)—suggested early cartridge replacement within the first 24 h to enhance cytokine modulation [5]. Subsequently, the FDA recommended a similar therapeutic scheme for managing cytokine storm in COVID-19 patients [6]. These experiences indicate that, depending on endogenous mediator production, earlier sorbent saturation may occur, thus requiring modulation of treatment intensity through adjustment of the cartridge replacement schedule. Notably, studies of HA in pediatric patients [7, 8]—in whom treatment intensity is generally higher than in adults—have reported more favorable outcomes than those observed in adult cohorts. Therefore, we aimed to explore the impact of treatment dose in a cohort of critically ill children with septic shock treated with HA, in order to better understand and identify potential key mechanisms underlying the therapeutic response to HA.

## Materials and methods

### Study design and population eligibility

We retrospectively reviewed all cases of pediatric septic shock who received HA with CytoSorb^®^ while receiving CRRT from January 2022 to December 2025. This study was approved by the Research Ethics Committee (REC) of the Children’s Hospital Bambino Gesù [ethical approval No. 136 (07/2023)]. Informed consent was obtained from the parents or the child’s next of kin. All study procedures complied with the ethical standards and guidelines of the REC for human experimentation and adhered to the principles of the 1975 Declaration of Helsinki and its subsequent amendments.

Pediatric septic shock was defined according to the Phoenix criteria [9]. Given the study period, Phoenix criteria were applied retrospectively for study classification to ensure consistency with current sepsis definitions. Clinical management was performed according to the latest guidelines [10]. AKI and its stages of severity were defined according to the Kidney Disease Improving Global Outcomes (KDIGO) criteria, and indications for CRRT were fluid overload, electrolyte imbalance, or both.

Hemoadsorption (HA) treatment with CytoSorb^®^ was applied as part of usual care in children admitted to PICU and was primarily physician-dependent, although predefined operational criteria were used in the PICU. HA was performed as an adjunctive therapy within 24 h after confirmed or suspected diagnosis of septic shock in children weighing ≥ 10 kg with need for CRRT. Treatment initiation was guided by clinical evolution during an observational period of up to 6 h under standard therapy. In particular, HA was initiated in patients showing signs of clinical deterioration, including increasing lactate levels, escalating vasopressor requirements, need for additional inotropic support, and/or evidence of sepsis-associated myocardial dysfunction (Additional file 1).

### CRRT and hemoadsorption with cytosorb

A hemodialysis catheter was inserted into a central vein (internal jugular or femoral), as appropriate according to patient size. CRRT was performed with standard hemofilters (polyarylethersulfone or AN69) combined with CytoSorb in continuous veno-venous hemofiltration (CVVH) or continuous veno-venous hemodiafiltration (CVVHDF) modality, using pre-filter reinfusion and an effluent dose of 2000 mL/h/1.73 m². Blood flow was selected according to body weight: ≤5 kg, 5–10 mL/min/kg; 5–10 kg, 5 mL/min/kg; >10 kg, 80–100 mL/min. CytoSorb was inserted in the CRRT circuit in series with the hemofilter in a post-filter position. Both the CRRT circuit and CytoSorb were flushed with saline solution and primed with albumin, blood, or saline at the discretion of the attending physicians. Anticoagulation was managed with regional citrate anticoagulation. In case of contraindications to citrate, continuous infusion of unfractionated heparin sodium (10–20 IU/kg/h) was used to achieve a post-filter activated clotting time (ACT) between 160 and 180 s. CytoSorb therapy was continued based on the clinical course as well as laboratory surrogates (lactate concentrations, metabolic status including pH). The adsorber was changed every 24 h as recommended by the manufacturer and continued for a maximum of 72 h.

### Clinical and surrogate endpoints

Clinical endpoints included hemodynamic improvement and mortality. Hemodynamic improvement was assessed using the Vasoactive–Inotropic Score (VIS), calculated immediately before HA initiation (defined as time 0, T0), after 24 h (T1), after 48 h (T2), after 72 h (T3) and exactly 24 h after the end of blood purification with CytoSorb^®^ (defined as Tend). Mortality was reported as PICU mortality and 28-day mortality. The surrogate endpoint was the biological response to HA, defined as the change in circulating interleukin-6 (IL-6) concentration during treatment. IL-6 concentration (pg/mL) was quantified immediately before HA initiation (defined as time 0, T0), after 24 h (T1), after 48 h (T2), after 72 h (T3) and exactly 24 h after the end of blood purification with CytoSorb^®^ (defined as Tend).

### Hemoadsorption dose and kinetic modeling

The amount of blood purified (ABP) was calculated as: ABP (L/kg) = Qb × T / (1000 × weight) [11], where Qb is blood flow (mL/min) and T is treatment duration (min). Extraction fraction (E) was defined as E = (Ci − Co) / Ci. Clearance was calculated as Cl = Qb × E. Assuming a one-compartment model with first-order kinetics, we defined the modeled removal fraction (MRF) as:

$$\rm MRF=1-exp (-E \times ABP / Vd)$$

To simulate progressive cartridge saturation, three representative extraction fractions were modeled: E = 0.99 (high), E = 0.2 (intermediate), and E = 0.05 (low), approximating Langmuir-type saturation kinetics [12]. These values were chosen for illustrative purposes and do not represent device-specific calibrated constants. Simulations were performed across a range of distribution volumes (Vd) from 0.5 to 50 L to evaluate whether higher Vd significantly impacted MRF.

We also calculated the observed removal ratio (RRAd%) for IL-6 as:

$$\rm RRAd(\%)=[(C_{T0}-C_{end})/C_{T0}]\times100$$

where C_T0_ is the baseline concentration (T0) and C_end_ is the concentration 24 h after the end of treatment (Tend) [13].

In three patients, in whom severe inflammatory burden allowed monitoring of cartridge saturation using inlet and outlet IL-6 concentrations across the hemoadsorber at 4 h (t_4hours_) and 8 h (t_8hours_) after the start of HA, a punctual estimation model was applied.

For these patients, mass balance (MB, in pg) over the interval was computed as:

$$\begin{aligned}&\rm MB(pg)=[(Ci(t_{4hours} )-Co(t_{4hours}))\\&+ \rm(Ci(t_{8hours})-C0(t_{8hours}))]/2\times Qb\\&\times \rm(t_{8hours}-t_{4hours})\end{aligned}$$

where Ci and Co represent concentrations pre- and post-removal at each interval, respectively, and Qb is the blood flow [14].

Given the high ABP delivered in our pediatric cohort, we hypothesized that, since pediatric patients receive an ABP dose exceeding the threshold value of 6 L/kg identified by Schultz, they may maintain elevated MRF values despite progressive column saturation (Langmuir effect). Furthermore, at comparable “supra-therapeutic” ABP levels, we explored which additional factors may influence removal capacity.

### Statistical analysis

Continuous variables are presented as median (IQR). Statistical significance was defined as *p* < 0.05. Analyses were performed using R (version 3.3.3) or SAS (version 9.4). To evaluate whether HA efficacy varied according to baseline inflammatory burden, cytokine removal was modeled as: Δlog(cytokine) = log(Cend) − log(C0), where C0 represents baseline concentration **(T0)** and Cend the concentration 24 h after the end of hemoadsorption treatment. Multivariable linear regression models included ABP (continuous variable), baseline log-transformed cytokine concentration, and an interaction term (ABP × baseline log cytokine). The interaction term tested whether delivered dose exerted differential biological effects at higher baseline mediator concentrations. Model performance was assessed using R² and adjusted R² values.

## Results

We consecutively enrolled 25 critically ill children treated with CytoSorb and CRRT for septic shock (Additional file 1 reports the study flow-chart). The overall median age in our population was 8 (5–13.5) years, and the overall median weight was 29 (16–60) kg. Additional file 2 included population’s characteristics. The median number of cartridges used per patient was 3, and in all the patients the duration of hemoadsorption was 72 h. Downtime of the extracorporeal circuit was not systematically recorded; however, no adverse events related to circuit clotting or interruption were reported during treatment.

### Schultz’s model in a pediatric setting

We divided patients’ weights into tertiles according to the sample distribution, obtaining the following clusters: the lowest (Cluster 1, *n* = 8) with a median weight of 15 (IQR: 14–16) kg, the intermediate (Cluster 2, *n* = 8) with a median weight of 28 (IQR: 23–31) kg, and the highest (Cluster 3, *n* = 9) with a median weight of 65 (IQR: 60–80) kg. After computing weight tertiles, we calculated the amount of blood purified (ABP), as defined by Schultz, in our cohort [11].

The median ABP (L/kg) in the overall population was 14.4 (IQR: 9.7–21.5). According to weight distribution, we found 23.5 (IQR: 17.2–28.8) L/kg in Cluster 1, 14.4 (IQR: 13.8–17.5) L/kg in Cluster 2, and 9.1 (IQR: 8.6–10.9) L/kg in Cluster 3. Considering that downtime of the extracorporeal circuits was not systematically recorded we acknowledge this is a potential source of ABP overestimation and potentially leading to an effective ABP slightly lower than calculated.

**Fig. 1:**
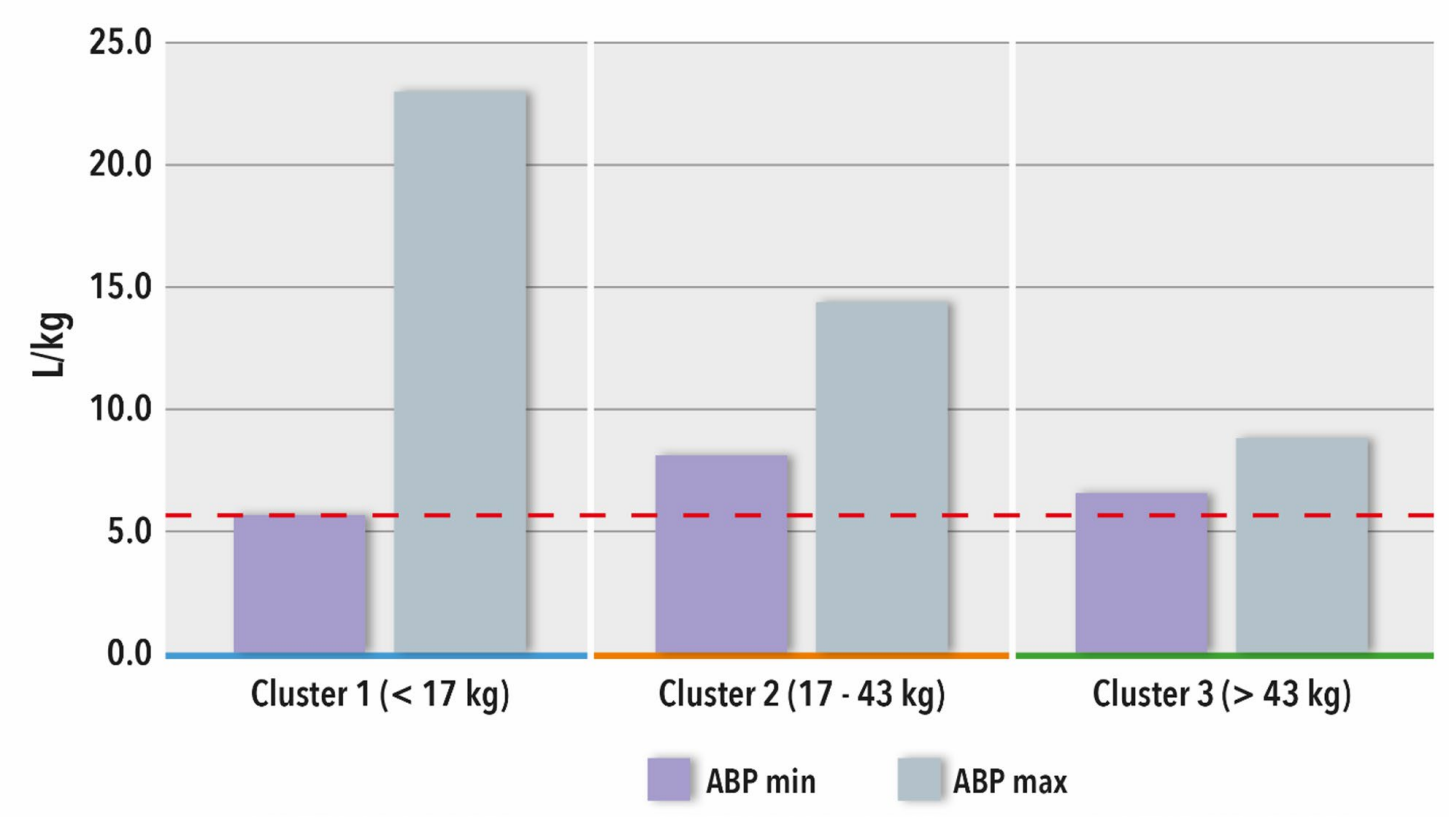
Median amount of blood purified in the three population weight clusters with maximal blood flow (ABP min) and minimal blood flow (ABP max). The red dotted line represents the 6 L/kg threshold according to Schultz [11]. L/kg = liters/kilogram

Figure 1 shows the median ABP and range values according to cluster weight and blood flow applied in the population. Table 1 shows the median ABP at different time points associated with cartridge exchange according to population clusters and absorber changes.

**Table 1:** The table reports the three clusters and the weight ranges

Cluster (weight, Kg)	*n*	Median weight	ABP 24 h	ABP 48 h	ABP 72 h
**Cluster 1 (Lowest** < 16)	8	15	7.4	14.8	22.3
**Cluster 2 (Intermediate** 16–42)	8	28	5.6	11.3	16.9
**Cluster 3 (Highest** > 42)	9	65	2.7	5.5	8.3

It includes the median weight and the median ABP at different time points, according to population clusters and absorber changes. *n* = number of patients

### Clinical biological response

Twenty patients had complete IL-6 measurements at baseline and at the end of HA therapy and were included in the analysis, while data were missing in five patients. Il-6 measurements were missing due to logistical and sampling-related issues rather than clinical characteristics with no apparent systematic pattern. To evaluate the potential impact of these missing data, a sensitivity analysis was performed. This analysis demonstrated that the study results, were robust to different assumptions regarding missing values.

In the present analysis, cytokine reduction was evaluated between baseline (T0) and 24 h after the end of hemoadsorption treatment (Tend). The removal ratio of IL-6 (T0-Tend) is shown in Table 2.

Beyond the median values, a marked variability was observed, as reflected by the wide interquartile ranges. This variability likely mirrors the substantial biological heterogeneity of the study population, despite similar allometric characteristics across the clusters.

**Fig. 2:**
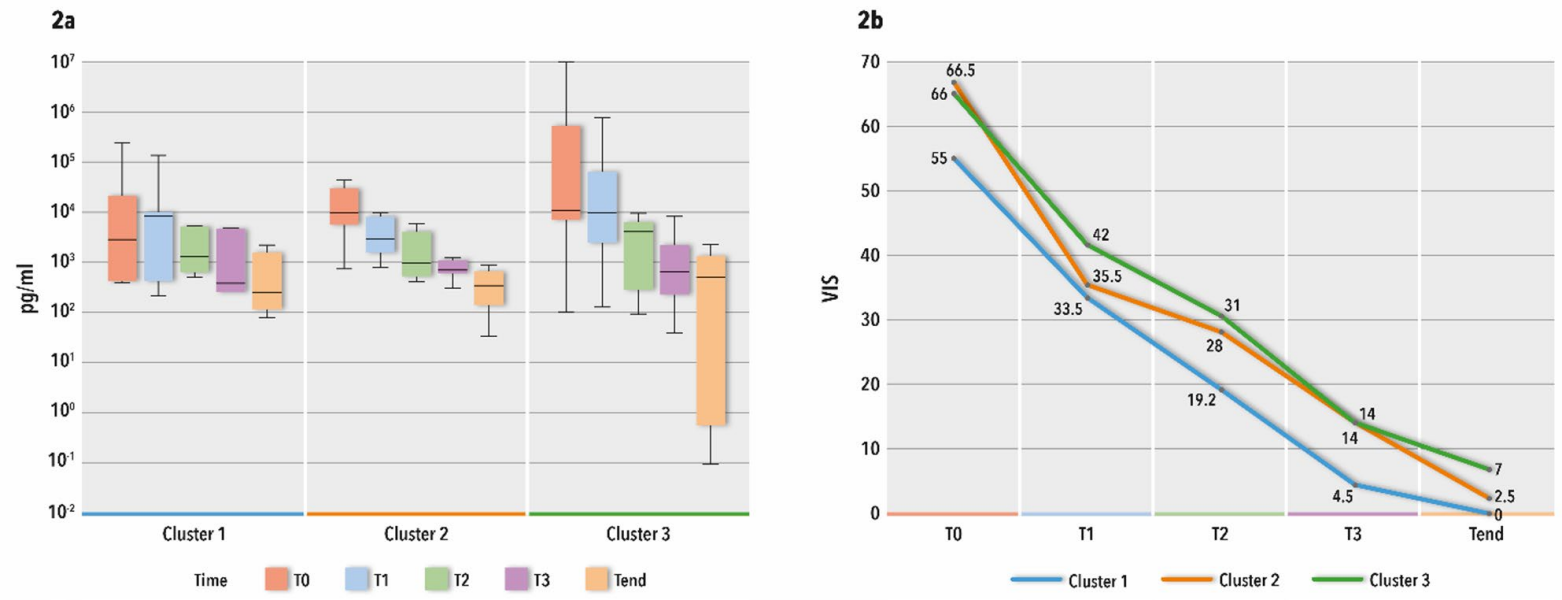
Panel **2a** shows the time course of interleukin-6 (IL-6) (pg/mL = picograms per milliliter) at different time points (T0 = baseline, T1 = 24 h after the onset of blood purification, T2 = 48 h after the onset of blood purification, T3 = 72 h after the onset of blood purification, Tend = 24 h after the end of blood purification). Panel **2b** shows the time course of the Vasoactive–Inotropic Score (VIS) at the same time points

Figure 2a shows the IL-6 time course at different time points during extracorporeal therapy with HA, according to weight clusters. Figure 2b shows the time course of the VIS score in the three population clusters. Additional file 3 reports the VIS score for each patient at each time point.

**Fig. 3:**
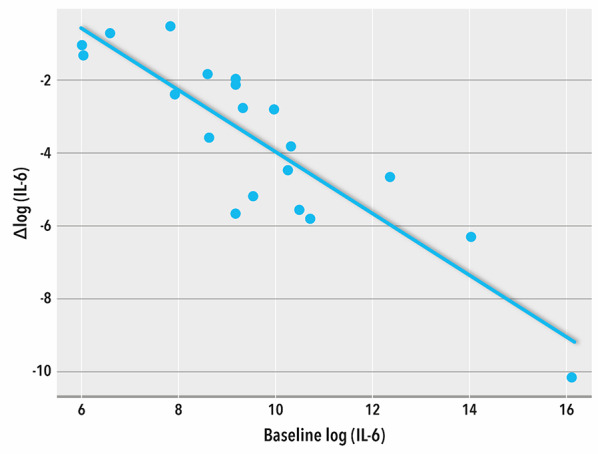
Graphical representation of the logarithmic decline of interleukin-6 (IL-6) according to baseline cytokine concentration

After log transformation, IL-6 reduction was modeled against delivered HA dose (ABP, L/kg) and baseline cytokine concentration. The multivariable interaction model demonstrated a strong overall fit (R² = 0.79, *p* < 0.001). Baseline log-transformed IL-6 concentration was independently associated with the magnitude of cytokine reduction (β = −1.15, 95%CI = [-1.08; -0.62], p = < 0.001), indicating greater logarithmic decline in patients with higher initial inflammatory burden (Fig. 3). In contrast, ABP was not independently associated with IL-6 reduction (*p* = 0.25), and no significant interaction between ABP and baseline IL-6 was observed (*p* = 0.24).

**Table 2:** The table reports the median removal ratio (RR%) and the median amount of blood purified and the median blood flow in the three clusters and baseline (T0) IL-6 median concentrations and IQR a across the three clusters

Clusters	ABP (L/kg)	Median Qb (ml/min)	Median IL-6 baseline (T0)	median RR% IL6 (T0-Tend)
**Cluster 1**	23.3	77,5	2.891 pg/ml (IQR1: 1.536 – IQR3: 18.367)	91%
**Cluster 2**	15.4	110	10.000 pg/ml (IQR1:5.773 – IQR3: 30.052)	98%
**Cluster 3**	9.4	125	10.792 pg/ml (IQR1: 9.144 – IQR3: 344.364)	100%

### Theoretical extra-corporeal removal capacity

**Fig. 4:**
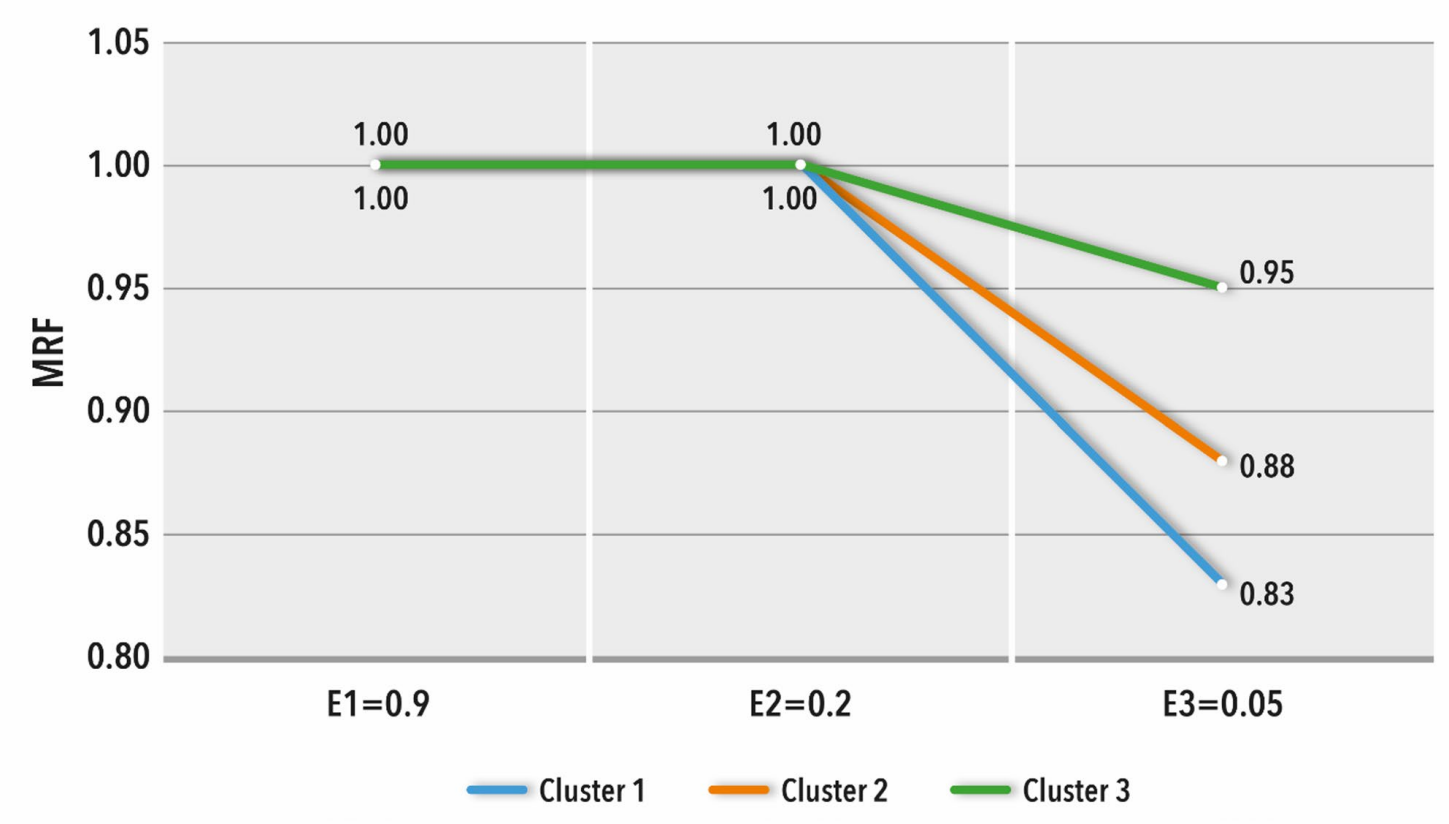
Graphical representation of the modeled removal fraction (MRF) for IL-6 in the pediatric population divided into three clusters (blue line: Cluster 1; orange line: Cluster 2; green line: Cluster 3), according to three representative extraction fractions: E = 0.99 (high), E = 0.2 (intermediate), and E = 0.05 (low)

**Fig. 5:**
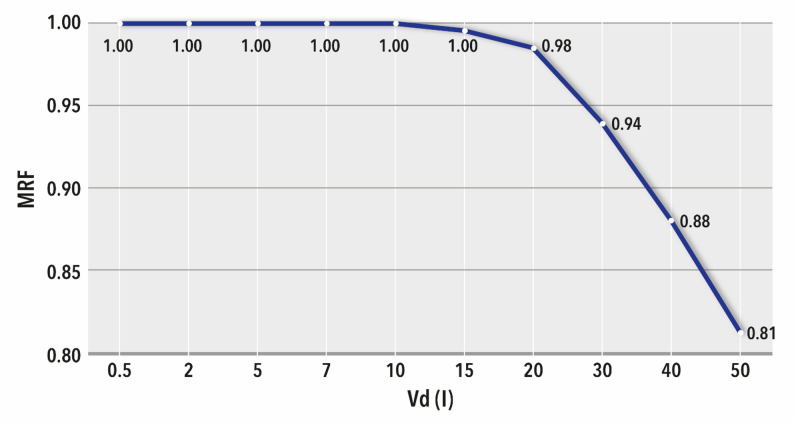
Graphical representation of the modeled removal fraction (MRF) for IL-6 (all clusters), selecting an intermediate extraction fraction (E = 0.2) and progressively increasing distribution volume (Vd)

The modeled removal fraction (MRF) according to ABP at a fixed Vd of 10 L, using three median extraction fractions (E1, E2, E3), is shown in Fig. 4. We then calculated the median IL-6 MRF in all clusters with progressively increasing Vd from 0.5 L to 50 L to evaluate whether higher Vd significantly impacted MRF, assuming E = 0.2 as the intermediate extraction fraction (Fig. 5).

**Fig. 6:**
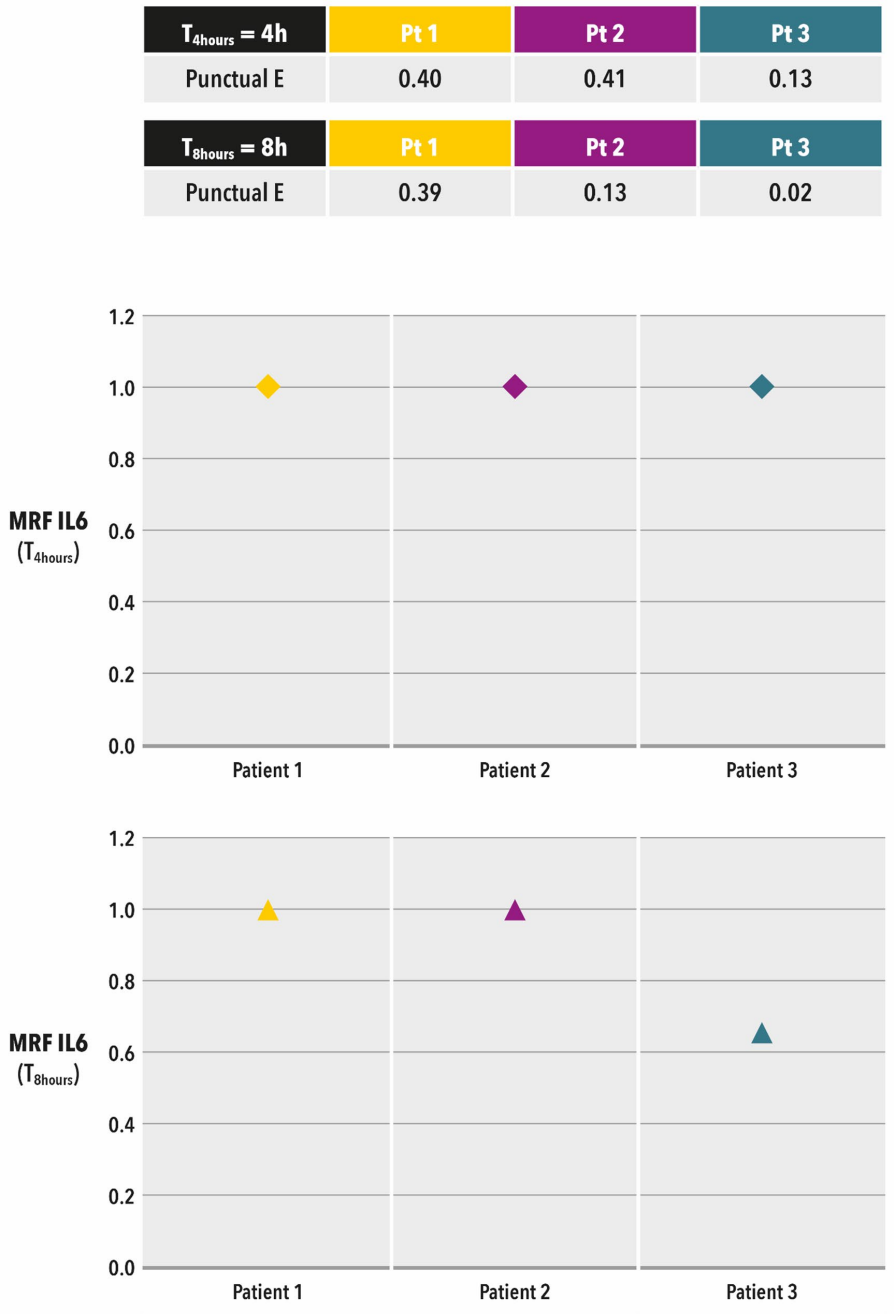
Graphical representation of the modeled removal fraction (MRF) for IL-6 in three patients using the punctual extraction fraction after 4 h from the onset of hemoadsorption (t_4hours_) and after 8 h (t_8hours_). The table at the top reports the exact values of the punctual extraction fraction in the three patients at t_4hours_ and t_8hours_

The punctual estimation of E for IL-6 in three patients, according to their ABP and with a fixed distribution volume of 10 L, is shown in Fig. 6. The point value E (punctual estimation) represents the extraction rate, calculated from pre- and post-sorbent samples at a given time point according to the formula $\:E=\frac{\mathrm{C}\mathrm{i}-\mathrm{C}\mathrm{o}}{\mathrm{C}\mathrm{i}}$.

**Fig. 7:**
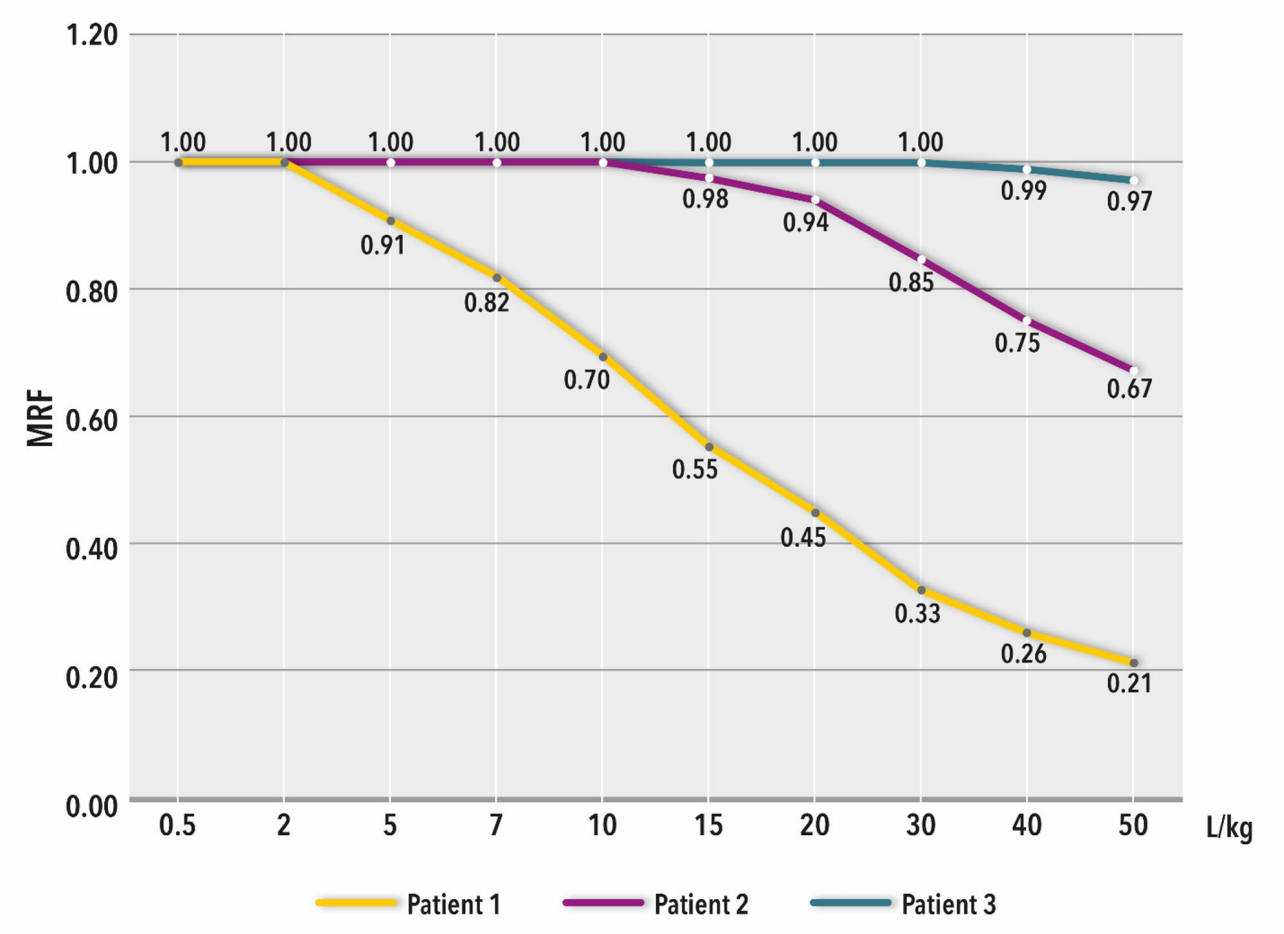
Graphical representation of the modeled removal fraction (MRF) for IL-6 in the three patients using the punctual extraction fraction after 8 h from the onset of hemoadsorption (t_8hours_) and progressively increasing distribution volume (Vd)

Figure 7 shows the MRF according to the punctual estimation of E at different Vd from 0.5 L to 50 L to evaluate whether higher Vd significantly impacted MRF.

### Direct circuit-based removal estimate (mass balance)

The initial IL-6 concentrations in patients 1, 2, and 3 were 8.806; 241.270; and 4.573 pg/mL, respectively. After 8 h of treatment with CytoSorb, a marked reduction was observed, with final values of 2.068; 119.950; and 2.965 pg/mL, respectively. Table 3 reports the mass balance in the three patients. The magnitude of removal is relevant (1,5 billion cumulatively removed), if we consider IL-6 concentrations is around 0–7 pg/ml in healthy patients with a pooled mean near 5 pg/mL [15]. In septic patients, IL-6 typically rises at least into the ten of pg/ml and often into the hundreds or thousands, especially in septic shock [3, 16].

**Table 3:** Mass balance values in the three patients for IL-6 between time point t4hours (4 h after the onset of hemoadsorption) and time point t8hours(8 h after the onset of hemoadsorption); pg = picograms

Patients	IL 6 Mass Balance 4–8 h (picogram)
Patient 1	58.116.000
Patient 2	1.409.280.000
Patient 3	9.144.000

## Discussion

In this exploratory study, we analyzed the concept of HA dose in critically ill children with septic shock treated with CytoSorb^®^ combined with CRRT. Our findings suggest that pediatric patients receive a substantially higher effective dose of HA when expressed as amount of blood purified per kilogram, compared with values typically reported in adult populations.

Recently, Berlot showed in a cohort of adult patients treated with CytoSorb^®^ better outcomes in terms of mortality and vasopressor reduction in those patients treated with higher intensity [17]. We observed in our cohort a survival in 22 out of 25 patients and a reduction of VIS score in our population. Using the ABP framework proposed by Schultz [11], all pediatric weight clusters in our cohort exceeded the threshold previously associated with improved survival in adults (6 L/kg), despite the use of lower absolute blood flows. This observation may support the hypothesis that, in pediatrics, delivered dose is consistently high and above adult thresholds, and this may contribute to the favorable hemodynamic trajectories observed in our cohort. However, given the retrospective, observational, and non-controlled design of the study, we cannot exclude that concomitant adjuvant therapies administered as part of routine clinical care, including immunoglobulins and corticosteroids, may have influenced the time course of the VIS score and other outcomes.

However, ABP alone does not account for the non-linear behavior of sorbent-based devices. HA is expected to be inherently time-dependent and limited by progressive saturation of binding sites within the cartridge. To address this limitation, we combined ABP with a simplified kinetic model incorporating variable extraction efficiencies to simulate different phases of absorber saturation. Across a wide range of assumed extraction fractions and distribution volumes, modeled removal rates remained relatively high across the tested conditions, suggesting that the delivered pediatric dose may be sufficient to partially compensate for declining absorption efficiency over time. Regarding the E values choice, as we saw that for our median ABP and weight data, maintaining a Vd of 10 L (given by the literature for IL-6), the MRF would always be 100% up to an E of 0.1, we have chosen 1; 0.2 and 0.05 to build a curve. Furthermore, we explored that with median weight, ABP and E held constant, a reduction in MRF would require a volume of distribution (Vd) of at least 20 L. This finding supports the potential effectiveness of this therapy in pediatric patients.

Given the concentration-dependent nature of sorbent-based adsorption, we specifically tested whether the relationship between delivered HA dose (ABP) and cytokine removal was modified by baseline mediator burden. Interaction models were therefore constructed to explore whether higher initial cytokine levels amplified the biological effect of delivered dose. The magnitude of IL-6 reduction was strongly associated with baseline cytokine concentration (R² = 0.79, *p* < 0.001), consistent with concentration-dependent adsorption kinetics. Delivered ABP did not independently predict cytokine reduction within the high-dose pediatric range. This pattern was also observed across weight clusters, where patients in Cluster 3, despite receiving lower ABP, showed the highest IL-6 removal ratios, likely reflecting higher baseline cytokine concentrations. This observation supports the hypothesis that the treatment may operate in a supra-therapeutic dose range where removal becomes more concentration-driven rather than dose-limited. Our findings differ from those reported by Schultz et al., in which patients with high ABP exhibited a distinctly severe pro-inflammatory state, characterized by the highest IL-6 levels among all identified clusters. However the same authors also reported that the decrease in IL-6 concentrations was consistent with the concentration- dependent adsorption kinetics of the adsorber [11].

Several limitations of this modeling approach should be acknowledged. First, circulating cytokine concentrations reflect the net balance between extracorporeal removal and endogenous production. Ongoing cytokine generation during septic shock may lead to an underestimation of true extracorporeal clearance when removal is inferred from systemic concentration changes alone. Second, we assumed a one-compartment model with first-order kinetics, which, although reasonable for intravascularly accessible mediators, does not capture complex tissue redistribution or delayed equilibration. Accordingly, systemic IL-6 reduction does not directly reflect extracorporeal removal, as circulating concentrations result from the dynamic balance between removal and ongoing endogenous production.

In addition, the extraction fractions used in the model were selected as representative values to approximate different phases of adsorption and were not derived from device-specific calibration. The adsorption process itself follows saturation kinetics that are better described by Langmuir-type isotherms, characterized by high initial affinity and a progressive decline in clearance as binding sites become occupied. While device-specific adsorption constants are not publicly available, our use of staged extraction fractions represents a pragmatic approximation of Langmuir-like behavior and avoids systematic overestimation of cumulative removal.

Finally, given the limited sample size, the interaction model may be prone to overfitting, and the observed associations should be interpreted with caution. These findings should therefore be considered exploratory and hypothesis-generating.

## Conclusion

Pediatric HA practice suggests that treatment intensity, when normalized to circulating blood volume, may be an important determinant of biological response. Children appear to receive relatively higher HA exposure per kilogram, which is associated with early and sustained mediator decline despite progressive cartridge saturation and ongoing endogenous cytokine production.

These findings support the hypothesis that optimization of HA strategies may benefit from moving beyond fixed treatment schedules and incorporating the underlying inflammatory burden. A cytokine kinetic–based approach, adapting treatment intensity to the magnitude and temporal profile of inflammation, may represent a more rational framework to translate biological effects into clinical benefit. Similar to antimicrobial therapeutic drug monitoring, serial assessment of IL-6 kinetics may allow treatment intensity to be tailored to the individual inflammatory trajectory intensity rather than predefined treatment durations. However, prospective studies are required to define optimal dosing strategies and to assess whether these concepts are applicable to adult populations.

## Supplementary Information

Below is the link to the electronic supplementary material.

Supplementary Material 1: Enrollment flowchart. The figure illustrates the inclusion and exclusion criteria of the enrolled population.

Supplementary Material 2: Population characteristics table. The table provides an overview of population characteristics, including demographics, comorbidities, sepsis-related features , organ support and severity of illness).

Supplementary Material 3: Vaso-Inotropic Score (VIS) time-course. The table provides VIS score for each patient at each time points (T0 = baseline, T1 = 24 hours after the onset of blood purification, T2 = 48 hours after the onset of blood purification, T3 = 72 hours after the onset of blood purification, Tend = 24 hours after the end of blood purification).

## Data Availability

The raw data supporting the conclusions of this article will be made available by the authors, without undue reservation.

## Abbreviations

ABP: Amount of Blood Purified
CRS: Cytokine Release Syndrome
ECMO: Extracorporeal Membrane Oxygenation
FDA: Food and Drugs Administration
kDA: KiloDalton
HA: Hemo-adsorption
IQR: interquartile range
MB: Mass Balance
MRF: Modeled Removal Fraction
PELOD-2: Paediatric Logistic Organ Dysfunction
PICU: Pediatric Intensive Care Unit
REC: research ethics committee
RR: Removal Rate
RRAd%: Removal Ratio
VIS: Vaso-Inotropic Score
