# Supplementary figures and images for: Exploratory dose modeling of hemoadsorption in pediatric septic shock
Source: Intensive Care Med Exp. 2026 Jun 22;14:79. doi: 10.1186/s40635-026-00933-1 (PMC13287285; doi:10.1186/s40635-026-00933-1)

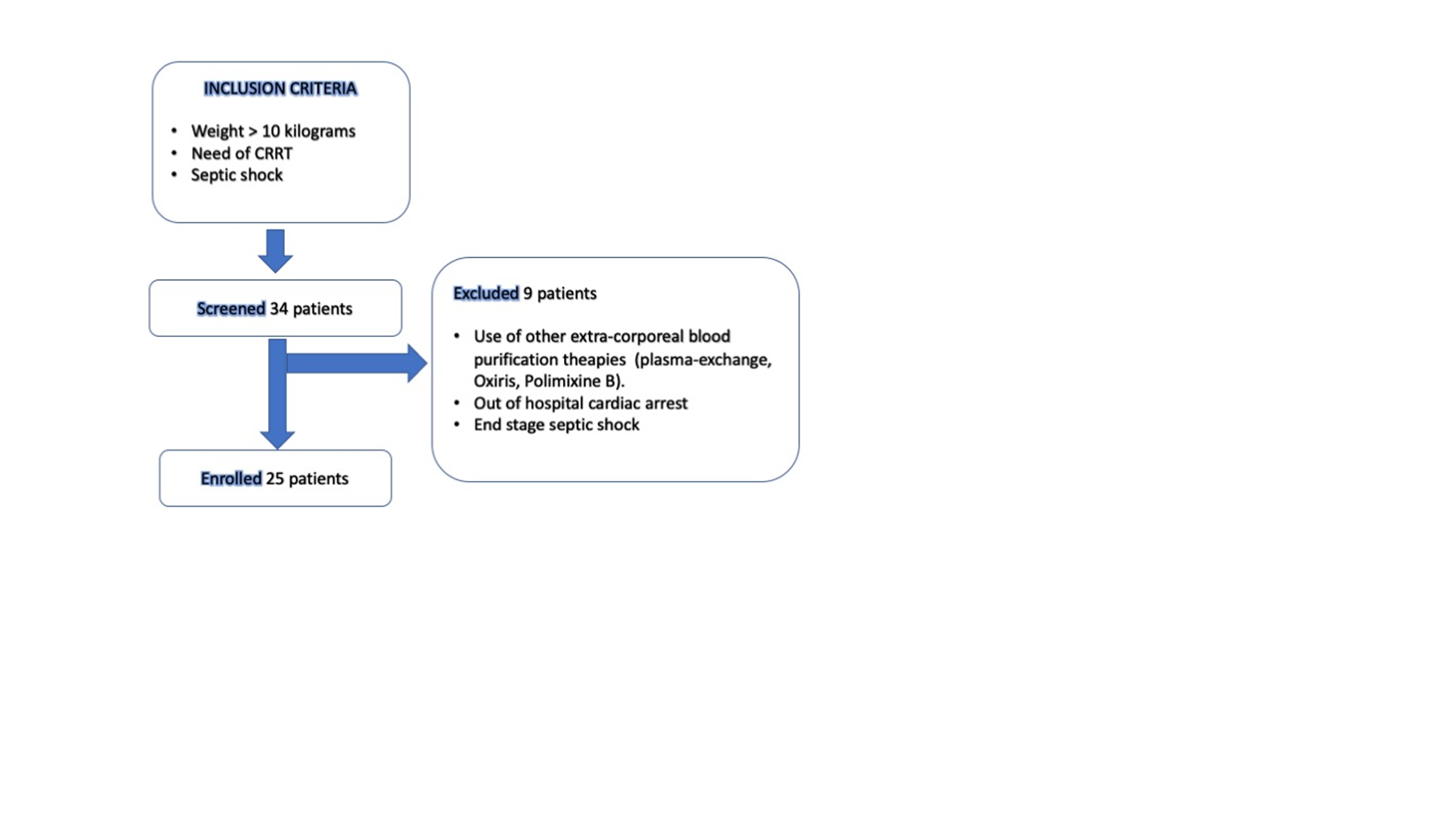

Supplement: Supplementary file 1 — Supplementary Material 1: Enrollment flowchart. The figure illustrates the inclusion and exclusion criteria of the enrolled population. [file 40635_2026_933_MOESM1_ESM.tif]
