# Supplementary material for: Exploratory dose modeling of hemoadsorption in pediatric septic shock
Source: Intensive Care Med Exp. 2026 Jun 22;14:79. doi: 10.1186/s40635-026-00933-1 (PMC13287285; doi:10.1186/s40635-026-00933-1)
Supplement: Supplementary file 2 — Supplementary Material 2: Population characteristics table. The table provides an overview of population characteristics, including demographics, comorbidities, sepsis-related features , organ support and severity of illness). [file 40635_2026_933_MOESM2_ESM.docx]

|  | **(n=25)** |
| --- | --- |
| Age, years | 9,2 [5,5-13,8] |
| Weight, kgs | 29 [16-60] |
| Male, n (%) | 13 (52) |
| PELOD-2 | 10 [7-11] |
| Hemato-oncological diseases | 8 |
| Post-surgical septic shock | 4 |
| Others | 13 |
| Source of infection   - Primary bacteremia - Respiratory - Abdominal - Meningo-encephalitis | 17  1  5  2 |
| Primary pathogen   - Bacteria (Gram positive) - Bacteria (Gram negative) - Virus - Fungus | 11  13  2  0 |
| Sedation, n (%) | 25 (100) |
| Invasive mechanical ventilation, n (%) | 25 (100) |
| PELOD-2 score | 10 [7-11] |
| VIS score | 65 [41-85] |

**Table 1:** Demographic characteristics of the population.

PELOD-2= Pediatric Logistic Organ Dysfunction 2; VIS= vasoactive inotropic score; NA = not available
