# Supplementary material for: Exploratory dose modeling of hemoadsorption in pediatric septic shock
Source: Intensive Care Med Exp. 2026 Jun 22;14:79. doi: 10.1186/s40635-026-00933-1 (PMC13287285; doi:10.1186/s40635-026-00933-1)
Supplement: Supplementary file 3 — Supplementary Material 3: Vaso-Inotropic Score (VIS) time-course. The table provides VIS score for each patient at each time points (T0 = baseline, T1 = 24 hours after the onset of blood purification, T2 = 48 hours after the onset of blood purification, T3 = 72 hours after the onset of blood purification, Tend = 24 hours after the end of blood purification). [file 40635_2026_933_MOESM3_ESM.docx]

|  | **VIS T0** | **VIS T1** | **VIS T2** | **VIS T3** | **VIS Tend** |
| --- | --- | --- | --- | --- | --- |
| Pts 1 | 41 | 20 | 13 | 6 | 0 |
| Pts 2 | 39 | 20 | 5 | 3 | 0 |
| Pts 3 | 70 | 30 | 4 | 0 | 0 |
| Pts 4 | 85 | 29 | 34 | 0 | 0 |
| Pts 5 | 45 | 45 | 25 | 12 | 13 |
| Pts 6 | 45 | 70 | 29 | 15 | 0 |
| Pts 7 | 65 | 65 | 37 | 24 | 5 |
| Pts 8 | 87 | 37 | 7 | 0 | 0 |
| Pts 9 | 53 | 33 | 24 | 28 | 39 |
| Pts 10 | 75 | 38 | 50 | 12 | 8 |
| Pts 11 | 19 | 17 | 15 | 5 | 0 |
| Pts 12 | 88 | 16 | 10 | 0 | 0 |
| Pts 13 | 0 | 0 | 0 | 0 | 0 |
| Pts 14 | 111 | 83 | 32 | 57 | 0 |
| Pts 15 | 75 | 75 | 65 | 41 | 9 |
| Pts 16 | 58 | 51 | 33 | 16 | 5 |
| Pts 17 | 71 | 32 | 22 | 14 | 0 |
| Pts 18 | 110 | 85 | 55 | 30 | 13 |
| Pts 19 | 27 | 14 | 16 | 10 | 5 |
| Pts 20 | 57 | 43 | 45 | 9 | 0 |
| Pts 21 | 24 | 23 | 19 | 6 | 0 |
| Pts 22 | 85 | 58 | 31 | 25 | 14 |
| Pts 23 | 65 | 80 | 33 | 17 | 17 |
| Pts 24 | 137 | 28 | 28 | 11 | 11 |
| Pts 25 | 27 | 42 | 48 | 18 | 7 |
